# Increased Glutamate in Somatosensory Cortex in Functional Dyspepsia

**DOI:** 10.1038/s41598-017-04405-1

**Published:** 2017-06-20

**Authors:** Arthur D. P. Mak, Georg Northoff, David K. W. Yeung, Winnie C. W. Chu, Steve C. N. Hui, Cynthia Cheung, Jessica Ching, Linda Lam, Sing Lee, Justin Wu

**Affiliations:** 1 Department of Psychiatry, Faculty of Medicine, The Chinese University of Hong Kong, Hong Kong, China; 2 Institute of Digestive Diseases, Faculty of Medicine, The Chinese University of Hong Kong, Hong Kong, China; 3 Department of Imaging and Interventional Radiology, Faculty of Medicine, The Chinese University of Hong Kong, Hong Kong, China; 4 Mind, Brain Imaging and Neuroethics Research Unit, University of Ottawa Institute of Mental Health Research, Ottawa, Canada

## Abstract

Functional Dyspepsia-Post-prandial Distress Syndrome (FD-PDS) was associated with *mood-related* increases in resting activity and lowered activation threshold in the somatosensory cortex (SSC), insula and perigenual anterior cingulate cortex(pgACC) in functional imaging studies. The underlying cortical neurochemical changes are unknown. We performed proton Magnetic Resonance Spectroscopy (1H-MRS) on 17 consecutive tertiary clinic-recruited psychotropic-naïve Rome III FD-PDS female and 17 age-sex matched healthy controls. Voxels were placed on bilateral pgACC, left insula and SSC. Water-suppressed spectra were acquired using PRESS with short echo time (TE) (T = 24 ms) to separately quantify glutamate (Glu) and glutamine (Gln). Main outcome measure was regional Glu/Cr + PCr. Severity of depression, anxiety, somatization, and dyspepsia were also assessed. We found significantly increased SSC Glu/Cr + PCr in FD-PDS subjects compared to controls. SSC Glu/Cr + PCr correlated significantly with postprandial distress chronicity, dyspeptic symptoms severity and anxiety. The SSC Glu/Cr + PCr - dyspepsia correlations became insignificant after controlling for anxiety but were independent of depression. Gln/Glu ratio, which indicates glial Glu cycling failure, was unchanged. No between-group differences were noted in other regional metabolite concentrations. Our findings suggested enhanced SSC glutamate transmission in FD-PDS that was linked to post-prandial distress chronicity and severity and anxiety.

## Introduction

Functional dyspepsia (FD) is characterized by chronic recurrent epigastric symptoms without organic diseases likely to explain the symptoms^1^. This functional gastrointestinal disorder (FGID) affects around 8%^2^ of the community, 81% being of the post-prandial subtype (FD-PDS)^2^, and a further 70% of them being female^3^.

Cross-sectional^2, 3^ and longitudinal^4^ population-based studies showed anxiety/depressive comorbidities to be common and interact bi-directionally with FD. Patients with FD and anxiety/depressive disorders are alike in their frequent somatic symptoms and preoccupation^5^. Peripheral etiologies aside (e.g. impaired gastric accommodation^6^, delayed gastric emptying^7^), FD has been linked to central factors such as hypersensitivity to gastric distension and duodenal contents^8^. Anxiety, depression and somatization, meanwhile, predicted dyspeptic symptom severity independent of gastric sensorimotor function^9^, suggesting the etiological significance of anxiety and depression in FD. The affective and somatosensory neurochemical pathways involved in FD are however insufficiently understood, belying the lack of a prevailing etiological model to guide effective treatment for FD.

Visceral sensory processing occurs via glutamatergic cortical networks. The **somatosensory cortex** (SSC) (Sensorimotor Network (SMN)^10^) receives spinothalamic visceral sensory input via the thalamus, and relays signals to the Salience Network (SN- **insula)**, the main cortical network processing autonomic, affective and interoceptive information^11^. SN sends control signals to (i) the *Default Mode Network* (DMN: midline structures including **perigenual anterior cingulate cortex (pgACC)**)^11^ to regulate *self-focus* and (ii) engage the Executive Network (EN: Dorsal-lateral prefrontal cortex (DLPFC)), which assigns *external* significance to sensory signals. Dysfunctions in these affective networks may impair gating of somatosensory signals^11, 12^.

In FD-PDS, structural magnetic resonance imaging (MRI) showed reduced ACC and insular grey matter density^13^ and microstructural changes in white matter tracts involved in somatosensory and affective processing^14^. Positron-emission tomography (PET)^15^ and functional MRI^16^ showed increased SN, DMN, thalamus and precuneus resting activity^16^, lowered SMN and SN activation threshold to gastric distension and failure of pain-related pgACC activation^17^. These correlated positively with dyspeptic^15^, anxiety and depressive symptoms^17^, suggesting *abnormal mood-related processing of somatic signals in the SMN, SN and DMN* ^17–19^.

Glutamate is the main cortical excitatory neurotransmitter^20^. Using magnetic resonance spectroscopy (MRS), a non-invasive MRI-based technique for discrimination and measurement of regional neurochemical concentrations, glutamate changes were reported in depression^21^ and anxiety disorders^22^. Reduced N-acetyl aspartate (NAA), which indicates neuronal integrity, and myo-Inositol (mI), which indicates glial proliferation and neuronal activity, were reported in depression^23, 24^. MRS on irritable bowel syndrome (IBS) showed reduced hippocampal glutamate, suggesting impaired HPA feedback^25^. Neurochemical changes in these networks is however unknown in FGIDs. Moreover, this study reported Glx, the combined concentration of glutamate and glutamine (Gln), which is difficult to interpret as Glu and Gln differentially vary with neuronal/glial physiology^26^. Reduced Gln/Glu ratio implies glial failure in the glutamine/glutamate cycle, whereas increased glutamate with normal Gln/Glu ratio represents enhanced neuronal glutamatergic transmission^27^.

Therefore, we set out to measure regional glutamate concentrations and Gln/Glu ratios in the SMN, SN and DMN in female FD-PDS subjects with *no prior psychotropic drug* treatment, versus age/sex-matched healthy controls. NAA and mI were measured to assess neuronal and glial integrity. We hypothesized glutamatergic abnormalities in SMN, SN and DMN in FD-PDS. We secondarily hypothesized correlation of regional glutamate concentrations with dyspeptic chronicity and severity. Correlations with general somatization, anxiety and depressive symptoms were also tested.

## Methods

### Recruitment

#### Subjects

We systematically identified 18 right-handed Chinese female FD-PDS subjects at a specialist gastrointestinal clinic. We only included female subjects to reduce sample heterogeneity introduced by gender effects, given the power limitations from the modest sample size, male being more prone to age-related brain metabolite changes^28^, and also considering that 70% of FD-PDS subjects in the community are female^3^. For recruitment, a gastroenterologist assessed the subjects to meet Rome III criteria^1^ for FD-PDS, with normal oesophago-gastroduodenoscopy results within the past 2 years. Gastrointestinal symptoms were rated using a Rome III-based questionnaire^29^ (see below). All eligible participants provided valid written informed consent. All experimental protocols were approved by the New Territories East Cluster- Chinese University of Hong Kong Clinical Research Ethics Committee and all procedures were performed in accordance with the approved guidelines and regulations.

For diagnostic ascertainment of anxiety and depressive-related disorders, as well as to exclude severe mental disorders, namely schizophrenia, delusional disorder, psychotic depression, bipolar disorder and substance use disorders, trained clinician interviewers performed a standardized assessment with the Chinese-Bilingual version of the Structured Clinical Interview for DSM IV-TR Mental Disorders-I^30^.

We excluded subjects who had (i) pregnancy or lactation, ii) consumed any of these drugs for currently and any lifetime 2-week period - antidepressants, neuroleptics, or drugs affecting gastrointestinal motility; (iii) history of gastrointestinal surgery or organic pathology that may explain dyspeptic symptoms (iv) chronic or severe medical conditions; (v) Evidence of severe mental illness including psychoses, bipolar disorder, or Mental retardation, organic brain syndromes or substance use; (vi) Had GERD or IBS as predominant symptom; (vi) Presence of any metallic materials in the body thus contraindicating MRI.

#### Control group

18 right-handed healthy female age-matched to the FD-PDS group were recruited via hospital posters and online advertisements, after a baseline assessment confirming eligibility according to recruitment criteria same as subject group except for history of mental or functional gastrointestinal disorders. FD-PDS and healthy control subjects were individual matched so that their age and years of education did not differ for more than 1 year apart.

### Assessment on the date of scanning

- A socio-demographic questionnaire detailing age, marital status, employment status, monthly family income, educational background.
- A structured Rome III symptom assessment questionnaire^29^ to assess current gastrointestinal symptoms. For dyspeptic symptoms, date of onset, frequency (past 3 months - never, <weekly, weekly, >weekly, everyday) and severity (past week − 0-asymptomatic; 1-mild, only recall on questioning and not affecting daily activity; 2-moderate, constantly aware of the symptom but not affecting daily activity; 3-severe, interferes with daily activity) of ‘bothersome postprandial fullness’, ‘early satiation’, ‘epigastric pain’ and ‘epigastric burning’ were recorded.
- Interviewer-administered Montgomery-Åsberg Depression Rating Scale^31^ (MADRS) for depression and Hamilton Anxiety Rating Scale (HAM-A)^32^ for anxiety.
- Chinese 15-item Patient Health Questionnaire (PHQ-15) - self-rated instrument for measuring general somatic symptom distress^33^.
- Chinese Short-form-36 health survey (SF-36) - generic self-rated instrument for assessing health-related quality of life^34^.
- Medication history - enquired and retrieved from hospital records.

### Magnetic Resonance Spectroscopy

Single voxel proton MR spectra were acquired using a 3-Tesla MRI scanner (Achieva TX series, Philips Healthcare, Best, Netherlands). Voxels were prescribed on a high-resolution T1-weighted 3D dataset acquired using a quadrature transmit/receive head coil in the transverse orientation from the same section of the brain (TR/TE: 7.4/3.4 ms, field of view: 250 × 250 mm, 285 contiguous slices, 0.6 mm (RL) thickness, reconstruction matrix: 240 × 240, flip angle 8°).

Signal transmission and reception for ^1^H-MRS were achieved using the quadrature head coil for higher B_1_ field and shorter TE. Water-suppressed spectra were acquired from each region using the point-resolved spectroscopic (PRESS) sequence (TR/TE 3000/24 ms). *The short TE PRESS sequence enabled optimal detection of Glutamate (Glu) peaks, which are prone to spectral overlap with glutamine peaks*.

Optimization procedures for spectroscopy consisted of automated receiver gain frequency adjustment, second-order shimming and gradient tuning. Water suppression was achieved using an automated selective inversion recovery technique. From each region, 128 water-suppressed signals were acquired at a spectral bandwidth of 1,000 Hz. The averaged signals were exported and processed on an off-line computer.

**Figure 1:**
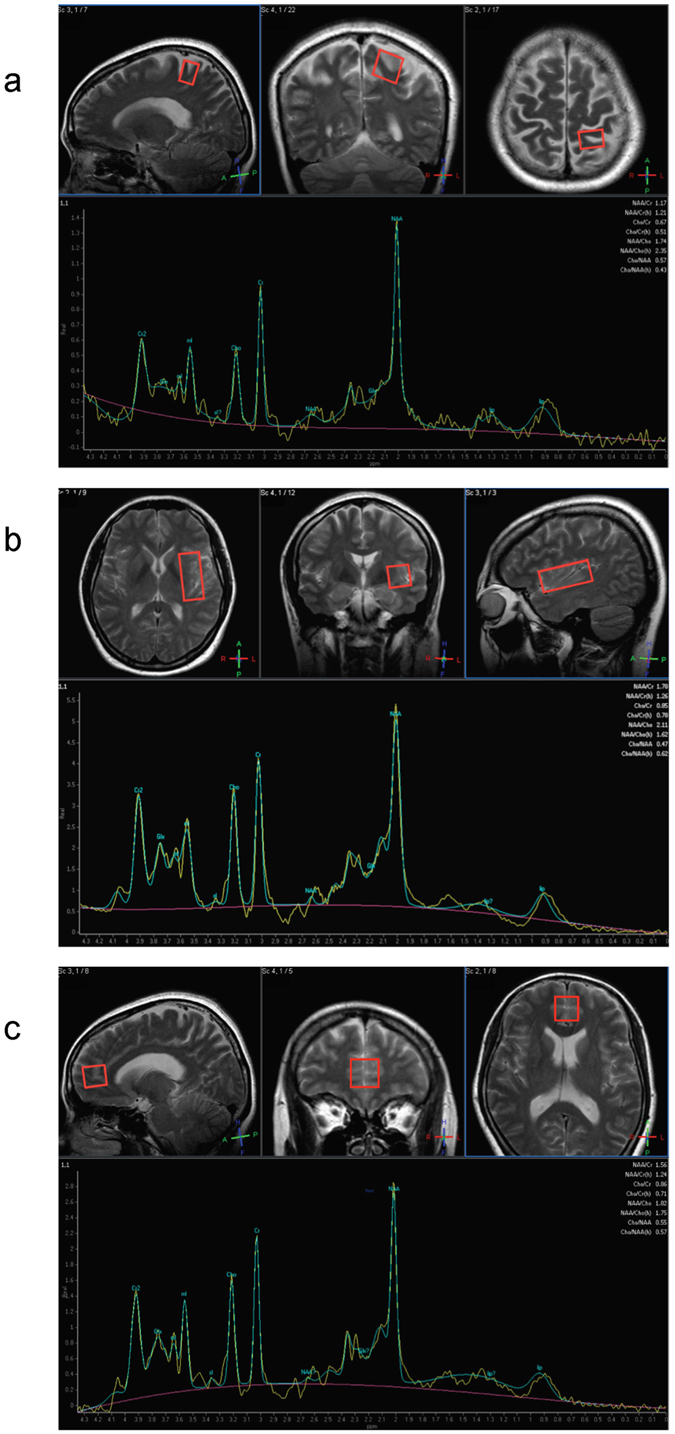
MRS Voxels of Interest (**a**). Left Somatosensory Cortex (20 mm × 15 mm × 20 mm) Voxels were placed on the left post-central gyrus (Brodmann areas 1, 2 and 3)^42^. (**b**) Left Insula (20 mm × 40 mm × 20 mm) Voxels were aligned along the edge of the insula cortex in an anterior-posterior direction with the anterior edge of the volume of interest aligned to the anterior limit of the insula^35^. (**c**) Bilateral pgACC (20 mm × 20 mm × 20 mm) represented the DMN, which interacts with SN to regulate self-focus, and shown to have reduced pain-related activation in FD-PDS^17^. Voxels were positioned as bordering the lower edge of the genu of the corpus callosum and with its posterior limits just touching the anterior border of the genu of the corpus callosum^56^.

Volumes of interests: (Fig. 1).

With the exception of pgACC, SSC and insula voxels were placed unilaterally side owing to time limitations. Previous functional neuroimaging studies on FD-PDS found bilateral alterations in these regions^15^. We selected the left side based on a previous study showing that left insula GABA and glutamate concentration to be correlated with interoceptive awareness, which is implicated in somatization^35^. (*Please refer to figure caption for details of voxel placemen-* Fig. 1).

### MRS Data Processing

Short TE spectra acquired were analyzed using a fully automated and user-independent spectral software package LCModel^36^ **(**Suppl. S1). Relative concentrations of Glutamate, NAA, mI to total creatine (Cr + PCr) were calculated based on peak concentration values obtained from LCModel analysis. Creatine is the most commonly used internal reference metabolite given the stability of its concentration^37^. No significant subject-control differences in creatine were found in any of the regions studied. Only results with adequate goodness-of-fit, as represented by the conventional criterion of the Cramer-Rao lower bound (CRLB) of 20 or less, were analyzed. Full-width maximum height (FWMH) was compared between the groups for each region by unpaired t-test to assess for differences in spectral quality.

#### Spectral quality measures

(Suppl. S2) The FD-PDS subjects and controls were comparable in their menstrual phase, regional gray matter/white matter ratios and FWHM in SSC, pgACC and insula. (Table 1) One FD-PDS subject had Glu/Cr + PCr CRLB > 20% in the left SSC and was excluded along with the respective matched control. All subjects and controls had Glu/Cr + PCr CRLB < 20% in pgACC and insula, and were included in the respective analyses. For other metabolite measures, FD-PDS subjects and controls were also excluded accordingly, thus the subject numbers included for each metabolite may differ.

**Table 1:** Between-group Comparison of Metabolite concentrations.

Brain Region	Metabolite	FD-PDS	Control	t	*Cohen’s d*	*p*
Left SSC	Glx/Cr + PCr (n = 16 vs 16)#	1.82 (0.19)	1.61 (0.12)	3.53	1.32	0.001*
	Glutamate/Cr + PCr (n = 16 vs 16) #	1.15 (0.11)	1.03 (0.07)	3.67	1.30	0.001*
	Gln/Glu ratio (n = 15 vs 15) #	0.59 (0.10)	0.58 (0.10)	0.18	0.07	0.86
	NAA/Cr + PCr (n = 17 vs 17) #	1.34 (0.10)	1.29 (0.12)	1.08	0.45	0.29
	mI/Cr + PCr (n = 17 vs17) #	0.65 (0.07)	0.63 (0.08)	0.60	0.27	0.55
Left insula	Glx/Cr + PCr (n = 17 vs 17) #	1.77 (0.20)	1.71 (0.08)	1.18	0.39	0.25
	Glutamate/Cr + PCr (n = 17 vs 17) #	1.11 (0.12)	1.06 (0.07)	1.47	0.51	0.15
	Gln/Glu ratio (n = 16 vs 16) #	0.61 (0.06)	0.61 (0.08)	−0.17	0	0.87
	NAA/Cr + PCr (n = 17 vs 17) #	1.19 (0.07)	1.19 (0.09)	0.14	0	0.89
	mI/Cr + PCr (n = 17 vs 17) #	0.71 (0.07)	0.68 (0.05)	1.49	0.49	0.15
Bilateral pgACC	GLx/Cr + PCr (n = 17 vs 17) #	1.92 (0.25)	1.83 (0.13)	1.33	0.45	0.19
	Glu/Cr + PCr (n = 17 vs 17) #	1.19 (0.12)	1.14 (0.10)	1.45	0.45	0.16
	Gln/Glu ratio (n = 17 vs 17) #	0.61 (0.10)	0.61 (0.08)	−0.07	0	0.94
	NAA/Cr + PCr (n = 17 vs 17) #	1.20 (0.08)	1.16 (0.10)	1.12	0.44	0.27
	mI/Cr + PCr (n = 17 vs 17) #	0.75 (0.07)	0.74 (0.08)	0.65	0.13	0.52

^*^Bonferroni corrected p < 0.003 (=0.05/(3 regions × 5 metabolite ratios)).

^#^Number of subjects included in analysis after removing those with Cramer-Rao Lower Bound ratio >20% for respective regional metabolite concentrations.

### Statistical analysis

The IBM SPSS version 22 was used for statistical analysis. Baseline demographic characteristics of the subjects were compared using unpaired t-test and Chi-square test for continuous variables and categorical variables respectively. Between-group differences in metabolite/Cr + PCr ratio in each region were tested with unpaired t-tests. For metabolites showing significant between-group differences, correlations with dyspeptic symptoms parameters (duration in years, current-week level of symptom distress and frequency of symptoms) were examined in FD-PDS subjects, and in both groups with mood symptom ratings (HAM-A, MADRS), and health-related quality of life (SF-36). The effect of anxiety and depressive comorbid diagnoses on any significant association between metabolite concentration and FD-PDS was also explored in multivariate analyses. A two-sided P-value of less than 0.05 was considered significant. For glutamate/Cr + PCr, Glx/Cr + PCr, glutamine to glutamate ratio, myo-inositol and NAA, we Bonferroni-corrected to type I error = 0.003(0.05/15) (15 referring to 3 regions × 5 metabolite ratios). Bonferroni correction was likewise used for multiple correlations (refer to respective results tables). Bootstrap corrections were applied to correct for potential outliers for significant correlations found.

## Results

### Demographic and clinical data

17 FD-PDS subjects and controls were analyzed, as one subject met criteria for Epigastric Pain Syndrome instead of FD-PDS by the date of scanning. The 2 groups were comparable in all sociodemographic variables. All FD-PDS subjects had active dyspeptic symptoms, generally of mild severity. Mean postprandial distress chronicity was 11.74 years. On average FD-PDS patients had mild-to-moderate HAM-A, MADRS and PHQ-15 scores, all significantly higher than controls. 9 FD-PDS subjects met DSM-IV-TR criteria for an anxiety disorder, while 3 met criteria for a depressive illness (Please refer to Suppl. S3). Physical-domain health-related quality of life (PCS) was significantly impaired in FD-PDS (Table 2).

**Table 2:** Demographic and Clinical Information in FD-PDS and Controls.

	FD-PDS (n = 17)	Control (n = 17)	*t/chi-square*	*p*
Sociodemographic				
Age, years (SD)	45.35 (9.59)	45.35 (10.01)	0.00	1.00
Education, years (SD)	11.82 (1.85)	11.53 (2.45)	0.40	0.70
Marital status, n (%)			1.54	
Married	11 (64.7)	12 (70.6)		0.67
Single	5 (29.4)	3 (17.6)		
Divorced	0	1 (5.9)		
Widowed	1 (5.9)	1 (5.9)		
Occupation status, n (%)			3.50	0.62
Full time	11 (45.8)	13 (76.5)		
part time	2 (11.8)	1 (5.9)		
unemployed	1 (5.9)	0		
student	1 (5.9)	0		
homemaker	2 (11.8)	2 (11.8)		
Retired	0	1 (5.9)		
Mood ratings				
Anxiety - HAM-A (SD)	17.29 (10.63)	2.24 (3.03)	5.62	<0.001*
Depression - MADRS (SD)	5.18 (8.06)	0.59 (1.33)	2.32	0.03*
Somatisation – PHQ-15 (SD)	8.76 (4.52)	3.29 (2.82)	4.23	<0.001*
Dyspeptic Symptoms				
Duration of symptoms, years (SD)	11.74 (12.3)	—	—	—
Frequency of dyspeptic symptoms				
Postprandial fullness, n (%)		—	—	—
None	0 (0)	—	—	—
<once a week	0 (0)	—	—	—
once a week or more	13 (76.5)	—	—	—
Every day	4 (23.5)	—		—
Early Satiety, n (%)		—	—	—
None	2 (11.8)	—	—	—
<once a week	1 (5.9)	—	—	—
once a week or more	14 (82.3)	—	—	—
Epigastric pain				
None	4 (23.5)	—	—	—
<once a week	6 (35.3)	—	—	—
once a week or more	7 (41.2)	—	—	—
Severity of Dyspeptic Symptoms				
Global dyspeptic symptoms (SD)	0.88 (0.78)	—	—	—
SF-36– physical component summary (SD)	47.05 (6.12)	51.85 (4.34)	−3.60	0.001*
SF-36- mental component summary (SD)	44.59 (11.95)	51.85 (9.40)	−1.97	0.06

*p < 0.05.

### Dyspeptic- Anxiety/Depression correlations

Anxiety (HAM-A) and depressive (MADRS) severity significantly correlated with post-prandial distress chronicity (HAM-A: r = 0.70, p < 0.001; MADRS: r = 0.65, p < 0.001), global dyspeptic symptom severity (HAM-A: r = 0.44, p = 0.01; MADRS: r = 0.56,p p = 0.001), current frequency of postprandial fullness (HAM-A: r = 0.67, p < 0.001; MADRS: r = 0.46,p = 0.01), early satiety (HAM-A: r = 0.53, p = 0.001; MADRS: r = 0.43, p = 0.01) and epigastric pain (HAM-A: p = 0.01; MADRS: p = 0.03), and were controlled for in the subsequent correlational analyses.

No significant difference was found in postprandial distress chronicity (13.7 vs 9.5 years, p = 0.50) and global dyspeptic severity (0.90 vs 0.90, t = −0.05, p = 0.97) in subjects with a DSM-IV-TR anxiety disorder versus those without. Subjects with a DSM-IV-TR depressive disorder had longer postprandial chronicity (24.5 years vs 9 years, t = −2.21, p = 0.04) but no significant difference in global dyspeptic symptom severity (1.33 vs 0.80, t = −1.11, p = 0.29).

### Metabolite concentrations

#### Between-group metabolite differences

**Figure 2:**
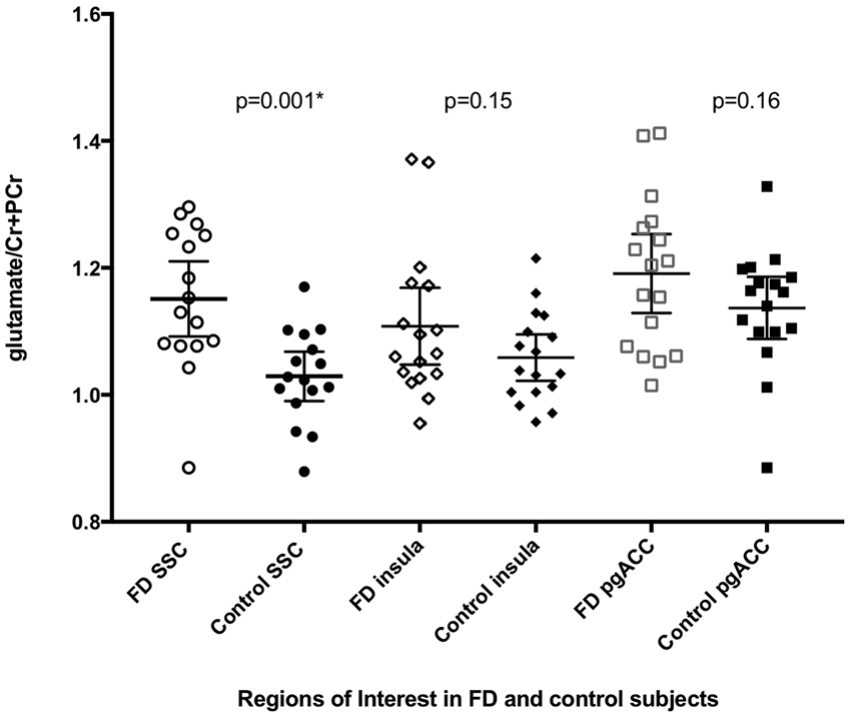
Glu/Cr + PCr of FD-PDS and controls in Left SSC, insula and bilateral pgACC. Glu/Cr + PCr = relative regional Glutamate concentration with total regional creatine + phosphocreatine as reference. SSC = somatosensory cortex, pgACC = perigenual anterior cingulate cortex. *p < 0.003 Bonferroni correction: p < 0.05/(3 regions × 5 metabolites).

(Figure 2 and Table 1) FD-PDS subjects had significantly higher SSC Glx/Cr + PCr (1.82(0.19) vs 1.61(0.12), t = 3.53, d = 1.32, p = 0.001) and Glu/Cr + PCr, (1.15(0.11) vs 1.03(0.07), t = 3.67, d = 1.30, p = 0.001) without significant difference in Gln/Glu ratio (0.59(0.10) vs 0.58(0.10), t = 0.18, d = 0.07, p = 0.86). Insula and pgACC Glx/Cr + PCr, Glu/Cr + PCr and Gln/Glu ratio were not elevated in FD-PDS. No changes were observed for mI/Cr + PCr and NAA/Cr + PCr in any of the regions.

Between-group SSC Glu/Cr + PCr difference remained significant after controlling for MADRS (p = 0.03), but became insignificant when controlling for HAM-A scores(p = 0.06), global dyspeptic symptom distress (p = 0.06), and post-prandial distress chronicity (p = 1.00) in binary logistic regression.

#### Glutamate – Dyspepsia correlations

**Figure 3:**
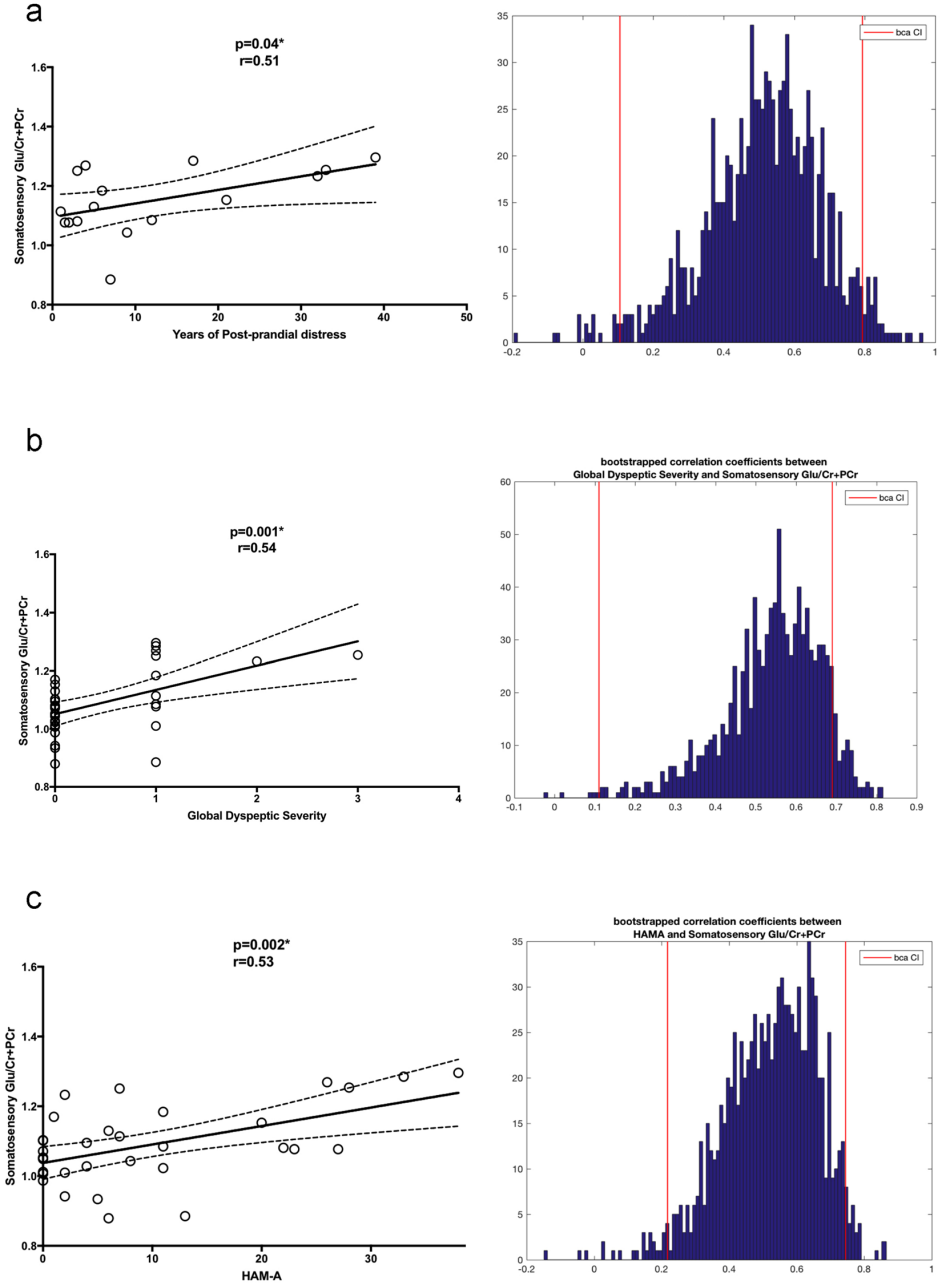
Somatosensory Cortex Glu and Clinical variable Correlations. Diagrams showing significant correlation between (**a**). Post-prandial Distress Chronicity (in years), (**b**). Global Dyspeptic symptom distress, and (**c**). Anxiety severity (HAM-A) with Somatosensory Glu/Cr + PCr ratio, accompanied with histograms of Bootstrapped correlation coefficients. Bias Corrected Accelerated (BCa) 95% CI is indicated by the red vertical lines. *Glu/Cr* + *PCr* = *relative regional Glutamate concentration with total regional creatine* + *phosphocreatine as reference*.

(Table 3 and Fig. 3) SSC Glu/Cr + PCr correlated significantly with post-prandial distress chronicity (r = 0.51, p = 0.04) in FD-PDS subjects. Across all subjects, it correlated significantly with frequency of postprandial fullness(r = 0.54, p = 0.001), early satiety(r = 0.48, p = 0.005) and epigastric pain(r = 0.47, p = 0.006), severity of early satiety (r = 0.54, p = 0.001) and global dyspeptic symptom distress (r = 0.54, p = 0.001). No significant correlation was found with general somatization (PHQ-15) (r = 0.20, p = 0.29). SSC Glu/Cr + PCr correlated significantly with anxiety (r = 0.53, p = 0.002, BCa 95% CI: 0.22–0.74). Correlation between SSC Glu/Cr + PCr and depressive severity became insignificant after bootstrapping (r = 0.45, p = 0.02, BCa 95% CI: −0.09–0.70).

**Table 3:** SSC Glutamate and Symptom Correlation in All Subjects.

Symptom	r	*p*	*BCa 95*% *CI*	
			*Lower*	*Upper*
Years of post-prandial distress	0.51	0.04*	0.11	0.79^+^
Frequency of symptoms				
*Postprandial fullness*	0.57	0.001^	0.29	0.78^+^
*Early Satiety*	0.48	0.005*^*	0.15	0.76^+^
*Epigastric pain*	0.47	0.006*^*	0.18	0.69^+^
Symptom distress over past 7 days				
*Epigastric pain*	0.43	0.01		
*Belching*	0.11	0.53		
*Epigastric burning*	0.12	0.53		
*Bloating*	0.28	0.12		
*Postprandial fullness*	0.44	0.01		
*Early satiety*	0.54	0.001^#^	0.33	0.72^+^
*Nausea*	0.40	0.02		
*Vomiting*	0.23	0.19		
Global dyspeptic symptoms	0.54	0.001*	0.11	0.79^+^
Mood ratings				
*HAM-A*	0.53	0.002*	0.22	0.74^+^
*MADRS*	0.45	0.01	−0.09	0.70
General Somatisation				
*PHQ-15*	0.20	0.29		

*p < 0.05.

^^^Bonferroni corrected p < 0.017 (=0.05/3).

^#^Bonferroni corrected p < 0.006 (=0.05/8).

^+^BCa 95% CI not including zero.

#### Somatosensory Glutamate – anxiety/depression – dyspepsia

(Suppl. S4) Correlations of SSC Glu(Cr + PCr) with postprandial distress chronicity (r = 0.64, Ba 95% CI: 0.19–0.78) and frequency remained significant after controlling for MADRS (r = 0.48, BCa 95% CI: 0.11–0.75) in partial correlation. Correlations of SSC Glu/Cr + PCr with all dyspeptic symptom variables became insignificant after controlling for HAM-A. Subjects with a DSM-IV-TR anxiety disorder did not show any significant difference in SSC Glu/Cr + PCr (1.17 vs 1.13, t = −074, p = 0.47), chronicity of postprandial distress 13.7 vs 10.3 years, t = −0.53, p = 0.60) or global dyspeptic severity (0.89 vs 0.86, t = −0.08, p = 0.94). In a linear regression model with SSC Glu/(Cr + PCr) as dependent variable, anxiety disorder and FD-PDS diagnoses as independent variables, FD-PDS independently predicted SSC Glu/Cr + PCr (p = 0.03, t = 2.30), but anxiety disorder did not (p = 0.39, t = 0.88). Likewise, depressive disorder did not significantly predict SSC Glu/Cr + PCr (p = 0.24, t = 1.19) in the linear regression model where FD-PDS independently predicted SSC Glu/Cr + PCr concentrations.

## Discussion

We found increased SSC glutamate in FD-PDS that correlated with dyspeptic severity and chronicity. Anxiety appeared to mediate the link between SSC glutamate and dyspeptic severity. To our knowledge, this is the first study measuring brain glutamate in FD-PDS, and to report glutamate and glutamine concentrations separately in FGIDs. Other strengths in our study include (i) closely matched FD-PDS and control groups (by age and education); (ii) all subjects were psychotropic drug-naïve; (iii) the FD-PDS subjects had mild-to-moderate dyspeptic, somatization, anxiety and depressive symptoms, which was representative of clinic patients.

Our findings are interesting in several ways. Firstly, increased SSC Glu but preserved Gln/Glu ratio suggested enhanced SSC glutamatergic *transmission* with intact Glu-Gln cycling in FD. Increased SSC Glu transmission may be compatible with fMRI findings of increased stimulus-dependent BOLD responses in SSC^18^, as previous studies had shown excitatory glutamatergic and inhibitory GABA-ergic transmission to be the neurochemical bases for stimulus-dependent BOLD responses^38^. We did not observe increase in pgACC and insula glutamate. SN GABA deficit, as described in depression^39^, may possibly underlie increased resting activity in FD-PDS^40^. The absence of changes in NAA and mI suggested preserved cortical neuronal integrity and glial proliferation in FD-PDS.

Secondly, the SSC glutamate - dyspeptic severity correlation is consistent with findings in other functional somatic syndromes (eg fibromyalgia, chronic pain) which showed the involvement of SSC glutamate in central pain sensitization^41, 42^. We did not observe any correlation between SSC glutamate and general somatization (PHQ-15), which may be related to the predominance of dyspeptic over other somatic symptoms in our subjects. Examination of the link of SSC glutamate with SMN structural and functional abnormalities in other FGIDs would further clarify the role of SSC glutamate in central visceral hypersensitivity - brain-related processes conferring enhanced perceptual responsiveness to visceral stimuli which are thought to underlie FGIDs^18, 43^.

The SSC glutamate - dyspeptic chronicity correlation is intriguing. Increased SSC glutamate with dyspeptic chronicity possibly reflected the consequence of neuroplastic and structural alterations from chronically increased viscero-sensory input from the gut, as shown in chronic pain and IBS^44, 45^, but could also be a result of early life adversities that predisposes to and increases chronicity of adult mental illnesses and functional somatic syndromes such as IBS and FD^10^. Although not measured in our study, early life adversities and chronic stress exposure are common in FGID patients ^12^. Early life stress in rat models was associated with enduring morphological changes and increased basal and stress-induced glutamate in the somatosensory cortex^46^. Other animal experiments showed chronic stress-induced glucocorticoid-mediated increase in prefrontal and hippocampal glutamatergic transmission and reduced glial proliferation that further impairs glutamate clearance^47^. While not yet examined in humans, reduced glutamate clearance and stress-related changes in glutamate release and receptor function were hypothesized to give rise to brain structural changes in stress-related psychiatric and functional somatic syndromes^48^. Intact SSC Gln/Glu ratio, mI and NAA in our FD-PDS subjects did suggest intact glial and neuronal integrity, but longitudinal imaging in subjects with well-documented trauma and stress exposure histories, and evaluation of stress-related biomarkers would be required to further clarify the pathophysiological context of SSC glutamatergic alterations in FD.

Thirdly, anxiety symptom severity correlated strongly with dyspeptic symptoms and mediated the association between SSC glutamate and dyspeptic chronicity and severity. While glutamate has been implicated in anxiety and depression^26, 49, 50^, the link between FD-PDS symptom/chronicity and SSC Glu was unlikely solely attributed to effects of a comorbid anxiety/depressive disorder, as FD-PDS predicted SSC Glu independent of anxiety/depressive diagnoses in our multivariate analysis. Rather, the impact on the SSC Glu-FD association from anxiety is on a *symptom level*. Anxiety symptoms have been known to have vast epidemiological, genetic, cognitive, and neural overlap with functional somatic syndromes^51^. In the only longitudinal population-based cohort study of FGID and anxiety/depressive symptoms, among individuals with no FGID at baseline, anxiety symptoms at baseline predicted subsequent onset of FGID (FD and IBS), suggesting an integral role of anxiety symptoms in the etiology of FD^4^. Our findings of anxiety symptom-mediated FD-PDS- SSC Glu correlation may be interpreted with the view of anxiety as an integral part of FD. This would then be consistent with other reports of anxiety-mediated alterations in structural connectivity^14^ and functional activity in SMN, SN and DMN reported in FD-PDS^17–19, 40^. Increased SSC glutamate transmission may in fact be the chemical basis of altered SMN, which along with altered SN and DMN activity, potentially underlie the anxiety-dyspepsia association in the community^2^.

No significant correlation between SSC glutamate and depressive severity was observed. Given the close FD-anxiety/depression inter-relationship^2^, the differential effects of anxiety and depression on SSC glutamate may suggest pathophysiological differences in the ways FD is linked to anxiety and depression. In the aforementioned population-based cohort study, while anxiety predicted future onset of FGID, it was depression but not anxiety at baseline that predicted persistence of FD in those with FD at baseline^4^. Anxiety and depression has been associated with increased glutamate in limbic, prefrontal and various other cortical areas^26, 49^, but few MRS studies on anxiety and depressive disorders have measured metabolites in the somatosensory cortex. Any differential somatosensory metabolite changes in anxiety and depressive disorders remains to be studied. On the other hand, the origin of the discrepancy could also be methodological, in that our sample contained more clinically anxious than depressed subjects. However, our analysis did not show any significant effect of an anxiety or depressive diagnosis on SSC glutamate. The specific effects of anxiety and depressive disorders on SSC Glu and dyspeptic symptoms should be examined by comparison with non-FD-PDS patients matched in mood ratings, and another group with only FD-PDS but no psychiatric morbidities, to clarify the specific effects of mood and anxiety disorders and FD-PDS.

Limitations in this study include firstly, that only 3 brain regions were measured. Costs limitations and the long acquisition time required to yield adequate signal-to-noise ratio for resolving glutamate peaks (15 minutes/region for 128 samples) necessitated the choice of representative regions in the SMN, SN and DMN, sacrificing other potentially relevant regions such as the DLPFC.

Secondly, the short-TE PRESS sequence could not resolve GABA peaks, as the small cortical quantities of GABA would yield a low signal-to noise ratio. Specific sequences required for resolving GABA, such as MEGAPRESS^52^, were not available at our unit at the time of study. GABA concentrations are related to insular and pgACC functional activity and its deficit is implicated in depression^39^. Measuring insular GABA in FD and depression would be important to explaining increased SN resting activity in the context of non-elevated glutamate.

Thirdly, we only included female subjects given the female preponderance in community FD-PDS samples^3^ and in view of sample size limitations, but may have different brain age-related chemical changes^28^, but this may also limit extrapolation of findings to male FD-PDS subjects. Future studies should preferably also examine SSC glutamate in male FD-PDS subjects.

Lastly, a larger sample would allow more power for insula and pgACC glutamate and other metabolite measurements, as well as stratified comparison of FD subjects with different psychiatric comorbidities, which would help understand the neurobiological basis of these brain-gut comorbidities.

## Conclusion

We found anxiety-related increase in somatosensory glutamatergic transmission in FD. Given the unsatisfactory treatment results of FD from monoaminergic agents^53^, our findings support exploration of glutamate as a novel therapeutic target. Drugs that reduce cortical glutamate excess (e.g. memantine and lamotrigine) have demonstrated efficacy in depression^54^, and physical exercise, which enhances glutamatergic synaptic plasticity and with proven efficacy for depressive and anxiety disorders^55^, should also be explored in treatment of FD.

## Electronic supplementary material

Supplementary Information
