# Supplementary material for: Increased Glutamate in Somatosensory Cortex in Functional Dyspepsia
Source: Sci Rep. 2017 Jun 20;7:3926. doi: 10.1038/s41598-017-04405-1 (PMC5478635; doi:10.1038/s41598-017-04405-1)
Supplement: Supplementary file 1 — Supplementary Information [file 41598_2017_4405_MOESM1_ESM.pdf]

Supplementary Information

Title: Increased Glutamate in Somatosensory Cortex in Functional Dyspepsia

Lead author: Arthur DP Mak

Co-authors: Georg Northoff, David KW Yeung, Winnie CW Chu, Steve CN Hui, Cynthia Cheung, Jessica Ching, Linda Lam, Sing Lee, Justin Wu

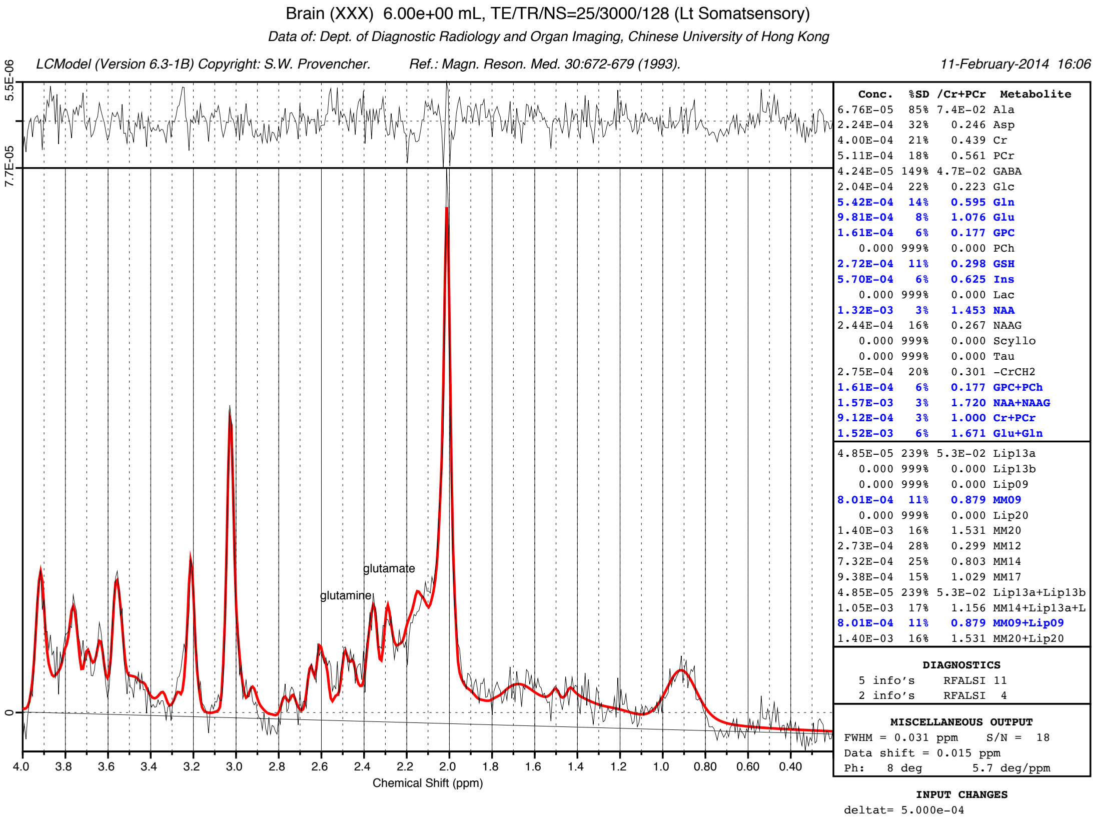

Suppl S1

Typical MRS spectra of a patient with FD-PDS

Suppl S2. Comparison of Regional GM/WM Ratio and Menstrual Phase between Patients with FD-PDS and Healthy Controls

	FD-PDS	Control	*P*
GM/WM ratio			
Left SSC	1.19(0.48)	1.13(0.22)	0.62
Bilateral pgACC	6.12(1.75)	5.27(1.46)	0.14
Left insula	2.13(0.47)	2.11(0.51)	0.91
FWMH			
Left SSC	0.04(0.01)	0.04(0.01)	0.72
Bilateral pgACC	0.04(0.01)	0.04(0.01)	0.49
Left insula	0.05(0.01)	0.05(0.01)	0.07
Menstrual phase			
Menstrual phase, no. of subjects(%)			0.78
follicular phase	6(35.3)	5(29.4)	
luteal phase	5(29.4)	4(23.5)	
menopausal	6(35.3)	8(47.1)	

- $p<0.05$

Suppl S3. DSM-IV TR anxiety and depressive diagnoses of FD-PDS subjects

9 subjects met criteria for an anxiety disorder

- 1 subject met criteria for Generalized anxiety disorder, social phobia and panic disorder with agoraphobia
- 1 subject met criteria for social phobia only
- 1 subject met criteria for agoraphobia without panic disorder
- 6 subjects met criteria for Generalized Anxiety Disorder

3 subjects met criteria for depressive and related disorders

- 2 subjects met criteria for a current Major Depressive Disorder
- 1 subject met criteria for Dysthymia.

Suppl S4 Partial Correlation of SSC Glutamate with FD variables controlled separately for Depression (MADRS) and Anxiety (HAMA)

		Control variables	
		MADRS	HAMA
postprandial distress duration	r	0.64 *	0.32
	BCa95% CI	0.19-0.78^{+}	-0.02-0.68
Frequency of postprandial fullness	r	0.48 *	0.24
	BCa95% CI	0.11-0.75^{+}	-0.14-0.64
Frequency of early satiety	r	0.26	0.02
	BCa95% CI	-0.21-0.65	-0.44-0.63
Frequency of epigastric pain	r	0.38	-0.11
	BCa95% CI	-0.004-0.62	-0.67-0.57
Global symptom distress	r	0.29	0.29
	BCa95% CI	-0.19-0.67	-0.15-0.64

*p< 0.05
$^{+}$BCa 95%CI not including zero

Legend: BCa 95% CI: Bias corrected accelerated 95% confidence interval
